# Persistence of inflammatory and vascular mediators 5 months after hospitalization with COVID-19 infection

**DOI:** 10.3389/fmed.2023.1056506

**Published:** 2023-02-10

**Authors:** James Melhorn, Asma Alamoudi, Alexander J. Mentzer, Emily Fraser, Anastasia Fries, Mark Philip Cassar, Andrew Kwok, Julian Charles Knight, Betty Raman, Nick P Talbot, Nayia Petousi

**Affiliations:** ^1^Nuffield Department of Clinical Medicine (NDM), University of Oxford, Oxford, United Kingdom; ^2^Oxford NIHR Biomedical Research Centre, University of Oxford, Oxford, United Kingdom; ^3^Department of Physiology, Anatomy and Genetics, University of Oxford, Oxford, United Kingdom; ^4^Wellcome Centre for Human Genetics, NDM, University of Oxford, Oxford, United Kingdom; ^5^Oxford University Hospitals (OUH) NHS Foundation Trust, Oxford, United Kingdom; ^6^Radcliffe Department of Medicine, University of Oxford, Oxford, United Kingdom; ^7^Chinese Academy of Medical Sciences Oxford Institute, University of Oxford, Oxford, United Kingdom

**Keywords:** COVID-19, inflammation, cytokines, vascular injury, post-COVID

## Abstract

**Background and aim:**

In acute severe COVID-19, patients present with lung inflammation and vascular injury, accompanied by an exaggerated cytokine response. In this study, our aim was to describe the inflammatory and vascular mediator profiles in patients who were previously hospitalized with COVID-19 pneumonitis, months after their recovery, and compare them with those in patients recovering from severe sepsis and in healthy controls.

**Methods:**

A total of 27 different cytokine, chemokine, vascular endothelial injury and angiogenic mediators were measured in the plasma of forty-nine patients 5.0 ± 1.9 (mean ± SD) months after they were hospitalized with COVID-19 pneumonia, eleven patients 5.4 ± 2.9 months after hospitalization with acute severe sepsis, and 18 healthy controls.

**Results:**

Compared with healthy controls, IL-6, TNFα, SAA, CRP, Tie-2, Flt1, and PIGF were significantly increased in the post-COVID group, and IL-7 and bFGF were significantly reduced. While IL-6, PIGF, and CRP were also significantly elevated in post-Sepsis patients compared to controls, the observed differences in TNFα, Tie-2, Flt-1, IL-7 and bFGF were unique to the post-COVID group. TNFα levels significantly correlated with the severity of acute COVID-19 illness (spearman’s r = 0.30, *p* < 0.05). Furthermore, in post-COVID patients, IL-6 and CRP were each strongly negatively correlated with gas transfer factor %predicted (spearman’s r = –0.51 and r = –0.57, respectively, *p* < 0.002) and positively correlated with computed tomography (CT) abnormality scores at recovery (r = 0.28 and r = 0.46, *p* < 0.05, respectively).

**Conclusion:**

A unique inflammatory and vascular endothelial damage mediator signature is found in plasma months following acute COVID-19 infection. Further research is required to determine its pathophysiological and clinical significance.

## Introduction

Clinical outcomes in acute coronavirus disease 2019 (COVID-19) are highly dependent upon the cytokine response in the host (1). The entry of SARS-Cov-2 virions into pulmonary epithelial cells *via* the angiotensin converting enzyme (ACE2), triggers a wave of pro-inflammatory cytokines and chemokines (2). In the healthy immune response infected cells are cleared and this inflammatory cascade recedes. In patients with more severe disease, however, an exaggerated elevation of these mediators has been observed, termed “cytokine release syndrome” (1, 3–5), which may lead to immunopathogenesis by causing tissue damage. These inflammatory pathways are the target of several successful treatments in the acute setting, such as dexamethasone and the anti-IL6R monoclonal antibody tocilizumab (6, 7). In addition, the vascular endothelium is also dysregulated in acute COVID-19 and microvascular thrombosis and endothelial inflammation contribute significantly to the pathology (8–10).

Contrary to the acute effects, our understanding of the longer-term effects of COVID-19 on inflammatory mediators and vascular function remains opaque, and other follow-up studies often have lacked an appropriate control group (11, 12). In this study, we examine levels of cytokine, chemokine and markers of vascular injury and angiogenesis in the peripheral blood of patients recovering from COVID-19 pneumonia many months after their acute infection, and compare their profiles to those of patients recovering from severe sepsis and to those of healthy controls.

## Methods

Our post-COVID cohort consisted of 49 patients [aged 60 ± 9 years (mean ± SD), 13 females] from whom venous blood was collected 5.0 ± 1.9 months after hospitalization with acute COVID-19 pneumonia. These patients were recruited from a post-COVID-19 follow-up respiratory clinic, having previously been hospitalized with acute COVID-19 pneumonia in the period between March 2020 and Jan 2021. For comparison, blood was obtained from a group of 11 patients 5.4 ± 2.9 months after hospitalization with severe sepsis (age 66 ± 17 years, seven females) (13) and 18 healthy control participants (age 47 ± 16 years, two females). Plasma was obtained by centrifugation of blood collected in EDTA-lined tubes and stored at –80°C prior to measurement of 27 different cytokine, chemokine, angiogenic and vascular injury markers [Meso Scale Discovery (MSD) V-PLEX multiplex assays using a Meso-Scale Discovery SQ120 device]: Interferon gamma, Interleukin 1B (IL-1B), IL-4, IL-6, IL-10, Tumor Necrosis Factor alpha (TNF alpha), Granulocyte-macrophage colony-stimulating factor (GM-CSF), IL-17A, Interleukin 12 (IL-12/23p40), IL-7, Macrophage Inflammatory protein 1A (MIP-1A), MIP-1B, Monocyte Chemoattractant Protein 1 (MCP-1), MCP-4, Interferon-inducible protein 10 (IP-10), Thymus and activation regulated chemokine (TARC), Vascular Endothelial Growth Factor A (VEGF-A), VEGF-C, VEGF-D, Placental Growth Factor (PIGF), Vascular Endothelial Growth Factor Receptor 1 (Flt-1), Angiopoietin 1 receptor (Tie-2), basic Fibroblast Growth Factor (bFGF), Serum Amyloid A protein (SAA), C-reactive protein (CRP), Vascular Cell Adhesion Molecule 1 (VCAM-1), Intercellular Adhesion Molecule 1 (ICAM-1) (see Table 1 for more details). All participants provided informed written consent. The studies were approved by the North-West Preston (20/NW/0235) and Oxford C (19/SC/0296) Research Ethics Committees.

**TABLE 1:** Multiplex assay results.

Plasma marker	Healthy pg/ml, (median, IQR)	Post-Sepsis pg/ml, (median, IQR)	Post-COVID pg/ml, (median, IQR)	Between three groups	COVID vs. Healthy	COVID vs. Sepsis	Sepsis vs. Healthy
**Pro-inflammatory**							
Interferon gamma	10.18 [7.81, 14.13]	10.24 [9.01, 14.68]	10.41 [7.50, 18.89]	ns			
IL-1B	0.049 [0.013, 0.160]	0.290 [0.074, 0.364]	0.104 [0.053, 0.141]	*p* < 0.05	ns	ns	+
IL-4	0.070 [0.044, 0.099]	0.082 [0.040, 0.112]	0.076 [0.050, 0.102]	ns			
IL-6	0.972 [0.696, 1.607]	2.264 [1.300, 4.058]	1.934 [1.295, 2.834]	*p* < 0.0005	+	ns	+
IL-10	0.654 [0.446, 0.798]	0.558 [0.442, 0.760]	0.630 [0.491, 0.941]	ns			
***TNF alpha***	***1.405 [0.754, 2.566]***	***1.616 [1.393, 1.938]***	***3.146 [2.109, 5.294]***	***p* < *0.0001***	**+**	**+**	***ns***
**Cytokine**							
GM-CSF	0.634 [0.329, 1.190]	0.409 [0.260, 1.244]	0.608 [0.340, 0.975]	ns			
IL-17A	6.366 [2.890, 13.330]	8.000 [4.521, 26.170]	8.015 [3.637, 16.320]	ns			
IL-12/23p40	283.1 [198.6, 344.3]	408.4 [297.1, 632.7]	325.0 [197.7, 478.4]	ns			
***IL-7***	***9.805 [6.689, 11.810]***	***7.785 [4.533, 33.360]***	***5.023 [3.263, 7.026]***	***p* < *0.005***	–	–	***ns***
**Chemokine**							
MIP-1beta	136.8 [110.3, 184.8]	229.9 [143.3, 336.7]	132.8 [97.8, 182.4]	*p* < 0.005	ns	–	ns
TARC	140.5 [112.5, 329.1]	340.7 [146.4, 550.3]	122.3 [81.7, 189.7]	*p* < 0.005	ns	–	ns
IP-10	585.9 [460.0, 820.1]	1,077.0 [455.7, 1325.0]	770.0 [520.3, 1117.0]	ns			
MIP-1alpha	50.39 [35.81, 335.20]	122.00 [48.67, 274.10]	45.43 [39.61, 57.28]	ns			
MCP-1	125.8 [184.5, 245.2]	208.5 [178.7, 328.8]	261.3 [214.7, 318.1]	ns			
MCP-4	119.2 [97.4, 155.5]	236.7 [125.6, 309.0]	134.3 [114.1, 184.2]	ns			
**Angiogenesis**							
VEGF-A	48.31 [34.02, 83.91]	76.94 [51.08, 152.20]	49.01 [30.09, 73.96]	ns			
VEGF-C	1129 [921, 1482]	1716 [1102, 2974]	1063 [782, 1668]	ns			
VEGF-D	726.8 [554.9, 944.3]	861.1 [702.8, 1127.0]	819.2 [684.4, 1092.0]	ns			
Tie-2*	***3434* ± *300***	***3245* ± *302***	***3923* ± *784***	***p* < *0.005***	**+**	**+**	***ns***
***Flt-1***	***93.49 [82.81, 109.60]***	***97.10 [65.96, 113.80]***	***124.40 [97.24, 157.90]***	***p* < *0.005***	**+**	**+**	***ns***
PIGF	3.010 [2.548, 3.177]	3.968 [2.577, 5.888]	4.260 [3.593, 5.360]	*p* < 0.0001	+	ns	+
***bFGF***	***22.17 [14.03, 41.34]***	***49.59 [9.19, 117.00]***	***2.06 [1.38, 4.23]***	***p* < *0.0001***	–	–	***ns***
**Vascular injury**							
SAA	1266741 [832641, 2592227]	2616109 [1540017, 8354912]	2720091 [1382324, 5395892]	*p* < 0.02	+	ns	ns
CRP	633598 [418901, 1559464]	3550592 [1632105, 6856682]	2253276 [1117127, 5794106]	*p* < 0.005	+	ns	+
VCAM-1	493133 [457268, 538105]	657406 [491048, 878164]	552729 [434409, 729479]	*p* < 0.05	ns	ns	+
ICAM-1	305599 [253191, 381896]	576237 [449555, 743289]	385068 [287088, 480021]	*p* < 0.005	ns	–	+

Five different MSD assay panels, each containing a set of mediators as shown, were used: (i) pro-inflammatory panel, (ii) cytokine panel (cytokines related to immune differentiation and growth), (iii) chemokine panel (cytokines related to monocyte chemotaxis), (iv) angiogenic, and (v) vascular injury panels (related to vascular injury and repair). Between-group analysis was performed by the Kruskal-Wallis test. Values are given as median and interquartile range (25th centile, 75th centile). Statistical significance was achieved if *p* < 0.05 and *p* < Benjamini-Hochberg critical value, with an FDR-adjusted q-value of 0.05. Where the three-way comparison between groups was significant, pairwise analysis was performed (Dunn test) for “post-COVID vs. healthy,” “post-COVID vs. post-sepsis,” and “post-sepsis vs. healthy.” + denotes significantly higher in 1st vs. 2nd group; – denotes significantly lower in 1st vs. 2nd group. Individual markers shown in bold and italic denote “COVID different from healthy control and different from post-Sepsis.” * for SAA, values are given as mean ± SD, and ANOVA test was used for between-group analysis (and Tukey’s test for pairwise comparisons) as data were normally distributed.

Statistical comparisons between groups were performed using Kruskal-Wallis tests for non-normally distributed data and one-way ANOVA for normally distributed data. To allow for multiple testing, false discovery rate (FDR) correction was performed using the Benjamini Hochberg method (FDR-adjusted q-value of 0.05). Where appropriate, pair-wise comparisons were undertaken using Dunn’s (or Tukey’s) multiple comparison tests. Associations between variables are given as Spearman correlation coefficients. Analyses were undertaken using Prism (version 8) and RStudio (version 1.2.5033).

## Results

Table 1 summarizes the assay results for all the mediators tested in the three participant groups, showing a three-way comparison between groups.

**FIGURE 1:**
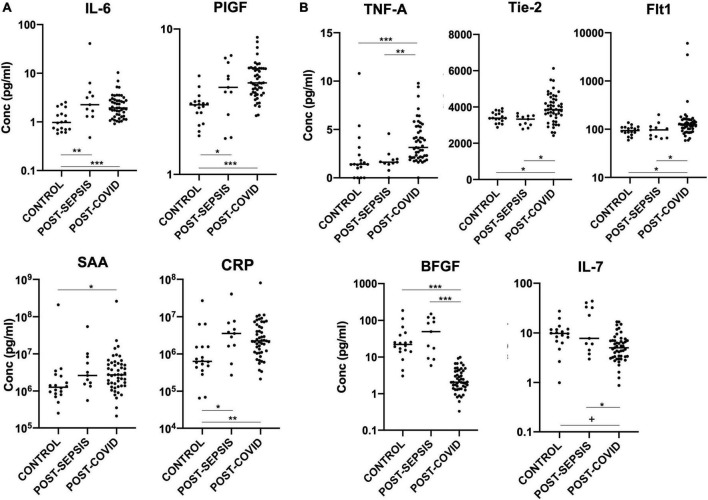
**(A)** Plasma markers elevated in both post-COVID and post-Sepsis groups, compared to healthy controls: IL-6, PIGF, SAA, and CRP. **(B)** Plasma markers uniquely different in post-COVID patients but not in post-Sepsis patients compared with healthy controls: TNFα, Tie-2, and Flt-1 are elevated while bFGF and IL-7 are depressed in post-COVID patients compared to healthy controls and compared to post-Sepsis patients. Horizontal bars indicate medians. +*p* < 0.01, *p < 0.05, **p < 0.005, ***p < 0.0005.

In keeping with previous reports of abnormal cytokine profiles months after COVID-19 infection (11, 12, 14), we demonstrate persistent significant elevation of IL-6, TNFα, Tie-2, Flt-1, PIGF, SAA, and CRP in our post-COVID cohort, compared with healthy controls, and persistent significant suppression of IL-7 and bFGF, as shown in Figure 1.

We are unable to determine whether these mediators are markers of previous disease in these patients or mediators of ongoing pathology. However, in a recent UK study of >2,000 patients, only 26% of patients felt fully recovered 5 months after COVID-19 infection, and IL-6 and CRP were among the cytokines persistently upregulated in those with more significant impairment (12). In our study, IL-6 and CRP levels in the post-COVID patients correlated positively with the thoracic computed tomography (CT) abnormality score (r = 0.28, *p* < 0.05 for IL-6, r = 0.46, *p* < 0.002 for CRP) and negatively with gas transfer factor (DLCO %predicted; r = –0.51, *p* < 0.002 for IL-6, r = –0.57, *p* < 0.0005 for CRP), which were performed at around the same time that blood was obtained for this study. Details of thoracic CT scores and lung function test results (DLCO and spirometry) are shown in Table 2. However, there was no correlation between breathlessness measured by the MRC dyspnea score (defined in Table 2) and levels of these or other measured mediators. Within the post-COVID group, we found a significant correlation between the severity of the acute illness (as defined in Table 2) and levels of TNFα (r = 0.30, *p* < 0.05).

**TABLE 2:** Severity scores of acute coronavirus disease-2019 (COVID-19) pneumonia and details of follow-up thoracic computed tomography (CT) and lung function tests in the post-COVID patients.

Severity of acute COVID-19 pneumonia	Severity = 0 (*n* = 24)	Severity = 1 (*n* = 14)	Severity = 2 (*n* = 11)
FEV1% predicted (Mean ± SD)	94.2 ± 26.5	93.3 ± 18.7	93.3 ± 19.3
FVC% predicted (Mean ± SD)	94.7 ± 23.3	96.7 ± 23.8	89.3 ± 17.7
DLCO% predicted (Mean ± SD)	76.5 ± 14.4	80.0 ± 14.8	73.8 ± 21.0
CT score No. of patients (%) 0 1 2	13 (54) 9 (38) 1 (4)	5 (36) 5 (36) 3 (21)	0 (0) 3 (27) 8 (73)
MRC dyspnea score Median, IQR	2 [1, 2]	2 [2, 2]	2 [1.5, 3]

Severity of acute coronavirus disease-2019 (COVID-19) pneumonia was classified according to requirement for respiratory support, where 0 indicates simple oxygen therapy, 1 indicated non-invasive respiratory support (e.g., continuous positive airway pressure or high-flow nasal oxygen therapy) and 2 indicates invasive mechanical ventilation. Lung function tests reported were performed at follow-up in the post-COVID clinic, around the same time that blood was obtained for this study. The forced expiratory volume in 1 s (FEV_1_), forced vital capacity (FVC) and gas transfer factor i.e., diffusing capacity of the lungs for carbon monoxide (DLCO) were expressed as a percentage of the predicted value, using the equations provide by the Global Lung Initiative. The CT thorax abnormality score was based on a review of CT imaging performed around the same time that blood was obtained for this study, and images were classified according to residual abnormality where 0 indicates normal images, 1 indicates mild residual ground glass change, and 2 indicates ground glass changes with additional lung fibrosis. Medical Research Council (MRC) Dyspnea Score is defined as: 1, not troubled by breathlessness except with strenuous exercise; 2, troubled by breathlessness when hurrying on the level or walking up a slight hill; 3, walks slower than most people of same age on the level because of breathlessness or has to stop for breath when walking at own pace on the level; 4, stops for breath after walking 100 yards or after a few minutes on the level; 5, too breathless to leave the house or breathless when dressing or undressing.

Importantly, our study also significantly adds to previous findings by identifying mediators for which expression is persistently abnormal in patients recovering from COVID-19, but not in those recovering from another pathology characterized by acute inflammation, namely severe sepsis. This group of mediators, which therefore constitutes a specific post-infection inflammatory/vascular injury signature of COVID-19, include the angiogenic factors Tie-2, Flt-1, and bFGF, and the inflammatory markers IL-7 and TNFα, as shown in Figure 1 and Table 1.

## Discussion

In this study we found persistent elevation of multiple mediators – IL-6, TNFα, SAA, Tie-2, Flt1, PIGF, and CRP – and persistent depression of IL-7 and bFGF in patients recovering from COVID-19 several months following their acute infection. Importantly, we also showed for the first time that persistent changes in Tie-2, Flt-1, bFGF, IL-7, and TNFα were uniquely seen in patients following recovery following COVID-19, but not in patients recovering from severe sepsis.

Our findings suggest that processes of endothelial injury and repair persist months after acute COVID-19 infection. The soluble angiopoietin 1 receptor (Tie-2), which has been previously reported increased following COVID-19 infection (11), is nearly exclusive to endothelial cells and has a critical role in antithrombotic signaling (15). Higher circulating levels of this receptor may reflect a homeostatic response to increased levels of its prothrombotic antagonist, angiopoietin-2 (15). Alternatively, it may reflect loss or cleavage of Tie-2 from the endothelial surface during ongoing inflammatory states. It could be a consequence of ongoing downregulation and shedding of angiotensin converting enzyme 2 (ACE2), the receptor for SARS-CoV-2, and a resulting accumulation of angiotensin 2 which has inflammatory and thrombotic effects when bound to the endothelial receptor AT1R (8, 16, 17). This final suggested mechanism may also explain our finding of increased levels of soluble vascular endothelial growth factor receptor (Flt1). Flt-1, an inhibitor of the vascular endothelial growth factor pathway which promotes endothelial dysfunction, has been shown to be increased acutely in COVID-19 (18) but its persistent elevation in the post-COVID setting is a novel finding of our study. The interaction between angiotensin 2 and the receptor AT1R has also been found to promote local Flt-1 upregulation in hypoxia (19). Therefore, dysregulation of the renin/angiotensin system is a potential unifying mechanism for our findings of increased Tie-2 and Flt-1 in post-COVID-19 patients. Unfortunately, we do not have data on tissue expression of ACE2, angiopoietin-2 or angiotensin 2 to explore this further.

Detrimental inflammatory and cytotoxic effects of angiotensin 2 binding to AT1R might also play a role in our finding of reduced levels of the basic fibroblastic growth factor (bFGF) in post-COVID-19 patients. BFGF is present in basement membranes, activated during wound healing, and has mitogenic effects on endothelial cells (20). Reduced levels of bFGF after COVID-19 could represent reduced production or increased consumption during healing from COVID-19 pneumonitis, or other endothelial injuries. This result, reported also by (11), shows an interesting contrast with the finding of elevated levels of bFGF in a large cohort of young adults with acute COVID who did not require hospitalization (21). It is possible that reduced levels of bFGF in our post-COVID-19 are a finding specific to more severe disease requiring hospitalization and post COVID-19 pneumonitis. It is noted, however, that no correlation is found here between reduced bFGF with raised inflammatory mediators IL-6 or CRP (which are associated with reduced gas transfer factor) or with TNF alpha (which correlates with severity of acute illness within our hospitalized cohort). Further exploration of the role and utility of bFGF in patients with post-COVID syndrome would be of interest.

We also found a persistent elevation of PIGF in post-COVID patients, which in the acute setting has been shown to correlate with in-hospital mortality (22), but did not see any differences (between COVID-19 patients and healthy controls) in levels of the vascular intercellular adhesion molecules ICAM-1 and VCAM-1 (important in inflammatory cell recruitment to the lung), nor in the endothelial growth factors VEGF-A, VEGF-C, or VEGF-D. The acute phase inflammatory proteins serum amyloid A and CRP are elevated in our post-COVID-19 cohort, in common with post-sepsis patients; but there are no differences in the levels of the leukocytic pyrogen and component of the inflammasome complex, IL-1β, from healthy control patients, in contrast with the findings of another study of patients reporting post-acute sequelae of COVID-19 (23).

The observed persistent suppression of IL-7 in our post-COVID cohort is also in keeping with previous reports (11), but contrasts the elevation of IL-7 in the acute setting (24, 25). IL-7 is critical for the development, maturation and survival of lymphocytes, and prevents memory cell apoptosis (26). In a recent study, recombinant IL-7 increased CD8+ and CD4+ T cell proliferation (*ex vivo*) in critically ill COVID-19 patients (27), and IL-7 has been suggested as immunotherapy and/or a vaccine adjuvant for COVID-19 (27–29). Reduced IL-7 after COVID-19 could reflect persistent lymphocyte exhaustion, contributing to inefficient viral clearance and chronic immune stimulation.

Tumor necrosis factor alpha, a pro-inflammatory cytokine, is elevated both in acute COVID-19 and acute sepsis and is linked to more severe disease and worse prognosis (4, 30, 31). In addition to its persistent elevation in our post-COVID patients, we identified a positive correlation between TNFα plasma levels after discharge and acute COVID-19 disease severity score (*p* < 0.05). No such correlation was identified for any other mediator in the current study. Others have found elevated TNFα in those with persistent symptoms many months after mild COVID-19 (14), and suggested it has a role in sustaining macrophage activation and cellular inflammation (14). These findings, as well the distinct TNFα elevation in post-COVID but not in post-Sepsis patients in our study, merit further study and consideration given the availability of established anti-TNF therapies.

Finally, of particular interest is the observation that elevation of IL-6 seen in our post-COVID cohort, and reported by others (11, 12, 14), is not specific to post-COVID but is also seen in a post-sepsis cohort. This has implications when considering anti-IL6 therapies in the post-COVID setting.

As noted above, a strength of our study is the control group consisting of patients recovering from sepsis, as well as a second group of healthy controls. Limitations include the relatively small numbers in the post-sepsis group, and the fact that patients were recruited early in the pandemic, prior to the emergence of later variants of the SARS-CoV-2 virus, such as the omicron variant. Notwithstanding these limitations, our key finding is that COVID-19 appears to be associated with a post-inflammatory signature that persists for at least 5 months, and that is distinct from the profile seen in patients recovering from sepsis. Understanding this signature may be important both for understanding the pathophysiology of the long-term effects of COVID-19, and for development or targeting of effective therapy. Further, more targeted study is required, for example in those who may suffer with prolonged symptoms (post-COVID syndrome), to understand the pathophysiological significance and potential clinical utility of theses uniquely persistent mediators.

## Data availability statement

The raw data supporting the conclusions of this article will be made available by the authors, without undue reservation.

## Ethics statement

The studies involving human participants were reviewed and approved by the North West Preston (20/NW/0235) and Oxford C (19/SC/0296) Research Ethics Committees. The patients/participants provided their written informed consent to participate in this study.

## Author contributions

BR, AM, NT, and NP conceived and designed the study. JM, AA, AM, AF, EF, AK, JK, and NP collected data. JM and NP performed experiments and analyzed data. JM, NT, and NP drafted the manuscript. All authors contributed to the interpretation of data, revision of the manuscript and approved the final version submitted for publication.
